# Isolated Ophthalmoplegia as an Atypical Manifestation of Miller Fisher Syndrome Post-viral Conjunctivitis

**DOI:** 10.7759/cureus.86170

**Published:** 2025-06-16

**Authors:** Christian John S Capirig, Celine Garcia, Gerard Francis Mangubat

**Affiliations:** 1 Internal Medicine, University of Hawaii John A. Burns School of Medicine, Honolulu, USA; 2 Internal Medicine, Jersey City Medical Center, Jersey City, USA; 3 Internal Medicine, Southern Philippines Medical Center, Davao City, PHL

**Keywords:** anti-gq1 antibody, atypical presentation, bilateral ophthalmoplegia, miller fisher syndrome (mfs), post-viral inflammation

## Abstract

Miller Fisher syndrome (MFS) is an uncommon variant of Guillain-Barré syndrome, typically identified by the presence of ophthalmoplegia, ataxia, and areflexia. While most cases follow this clinical pattern, atypical presentations lacking one or more components can complicate diagnosis. We report the case of an 81-year-old male with well-controlled hypertension who presented with acute-onset horizontal diplopia two weeks after a self-limiting episode of viral conjunctivitis. Neurological examination revealed complete bilateral ophthalmoplegia with preserved pupillary reflexes and no other focal deficits. Brain imaging and CSF analysis were unremarkable, with no evidence of albuminocytologic dissociation. A comprehensive serologic and autoimmune workup was negative, except for markedly elevated anti-GQ1b IgG titers (>1:12,800). The patient was diagnosed with an atypical form of MFS and received a five-day course of IVIG, which led to complete symptom resolution within one week. This case underscores the importance of considering MFS even in the absence of its full clinical triad. Anti-GQ1b IgG seropositivity remains a key diagnostic marker, and early treatment with IVIG can result in rapid and complete recovery. Clinicians should maintain a high index of suspicion for atypical MFS presentations, especially in patients presenting with isolated cranial nerve findings following a viral illness.

## Introduction

Miller Fisher syndrome (MFS) is a rare, immune-mediated variant of Guillain-Barré syndrome (GBS), first described by Charles Miller Fisher in 1956. It is classically characterized by a triad of ophthalmoplegia, ataxia, and areflexia [1]. Although MFS accounts for only 1-5% of GBS cases worldwide, it appears to be more prevalent in East Asian populations, with an estimated annual incidence of approximately 0.09 per 100,000 individuals [2].

The diagnosis of MFS is primarily clinical, based on characteristic neurological findings, and is further supported by specific serologic markers [3,4]. A key diagnostic indicator is the presence of anti-GQ1b IgG antibodies, which are detected in more than 85-90% of cases and reflect the syndrome’s underlying autoimmune mechanism [5]. Ancillary testing often includes CSF analysis, which may reveal albuminocytologic dissociation, elevated protein levels with a normal white cell count, as well as neuroimaging to exclude structural or vascular causes [2,4]. However, these findings may be absent in the early stages or in atypical variants of the disease [4,6].

We report a rare and diagnostically challenging case of atypical MFS, presenting as isolated ophthalmoplegia without accompanying ataxia or areflexia, following an episode of acute viral conjunctivitis. Despite the absence of the full clinical triad, the diagnosis was strongly supported by markedly elevated titers of anti-GQ1b IgG antibodies [5]. Extensive evaluation, including neuroimaging and CSF analysis, revealed no other abnormalities.

This case highlights the importance of maintaining a high index of suspicion for atypical MFS presentations, especially in patients with isolated cranial nerve deficits following recent viral illness. It also underscores the diagnostic utility of anti-GQ1b antibody testing in identifying MFS even in the absence of classic clinical features.

## Case presentation

An 81-year-old male with a history of well-controlled hypertension presented to his primary care clinic with a two-day history of acute-onset horizontal diplopia. The diplopia was constant, binocular, and worsened on lateral gaze. He denied associated symptoms such as headache, vision loss, eye pain, photophobia, vertigo, dysphagia, nausea, vomiting, or limb weakness. Notably, he had experienced an episode of viral conjunctivitis approximately two weeks earlier, initially involving unilateral eye redness, tearing, and irritation, which progressed to bilateral involvement. These symptoms were accompanied by mild upper respiratory tract features, including rhinorrhea, sore throat, and low-grade fever, all of which resolved spontaneously within several days. He remained symptom-free until the onset of diplopia.

Given the acute cranial nerve involvement, he was referred to the emergency department for further evaluation to rule out intracranial pathology. His medical history was otherwise unremarkable, aside from hypertension managed with amlodipine 10 mg daily. He had no history of diabetes, cardiovascular disease, autoimmune conditions, or prior neurologic events. His surgical history was notable only for an appendectomy many years earlier. He was not taking any recent antibiotics or over-the-counter medications and denied any known drug allergies. He lived independently with his spouse, had retired from a career in teaching, and denied alcohol, tobacco, or illicit drug use. There was no recent travel, no known sick contacts, and no relevant family history of neurologic or autoimmune disease.

On physical examination, he was alert, oriented, and hemodynamically stable. Neurologic examination revealed complete bilateral ophthalmoplegia affecting all extraocular movements. His pupils were equal, round, and reactive to light, with preserved accommodation and no ptosis or anisocoria. Fundoscopic examination was normal, and there was no evidence of papilledema. Cranial nerves, aside from those controlling eye movement, were intact. His motor strength, reflexes, cerebellar testing, gait, and sensory examination were all within normal limits.

A comprehensive laboratory evaluation was conducted to investigate autoimmune, infectious, metabolic, and paraneoplastic causes of isolated ophthalmoplegia (Table 1, Table 2). CBC revealed only mild relative elevations in neutrophils and monocytes. CRP was mildly elevated at 11.7 mg/L (reference: 0-10), though this was nonspecific. Testing for rapid plasma reagin, cryoglobulins, hepatitis B and C, HIV, antinuclear antibody (ANA), antineutrophil cytoplasmic antibody, neuromyelitis optica/aquaporin 4 fluorescence-activated cell sorting, and myelin oligodendrocyte glycoprotein fluorescence-activated cell sorting returned negative results. Levels of vitamin B1, B12, thyroid-stimulating hormone, angiotensin-converting enzyme, and serum complements were within normal limits. Paraneoplastic panels and heavy metal screens were also unremarkable.

**Table 1:** CBC results MCH, mean corpuscular hemoglobin; MCHC, mean corpuscular hemoglobin concentration; MCV, mean corpuscular volume; RDW, red cell distribution width

Test	Result	Reference range	Units
WBC count	10.31	3.80-10.80	× 10³/µL
RBC count	5.09	4.00-6.20	× 10⁶/µL
Hemoglobin	14.1	13.7-17.5	g/dL
Hematocrit	44	40.1-51.0	%
MCV	86.4	79.4-98.4	fL
MCH	27.7	26.0-34.0	pg
MCHC	32	32.0-36.0	g/dL
RDW	13.9	11.6-14.4	%
Platelet count	254	151-424	× 10³/µL
Absolute neutrophils	6.51 ↑	1.56-6.20	× 10³/µL
Absolute monocytes	0.95 ↑	0.24-0.82	× 10³/µL

**Table 2:** Comprehensive metabolic panel results ALT, alanine aminotransferase; AST, aspartate aminotransferase; BUN, blood urea nitrogen; CKD-EPI, Chronic Kidney Disease Epidemiology Collaboration; eGFR, estimated glomerular filtration rate; SGOT, serum glutamic oxaloacetic transaminase; SGPT, serum glutamic pyruvic transaminase

Test	Result	Reference range	Units
Glucose	137 ↑	70-99	mg/dL
BUN	20	6-23	mg/dL
Creatinine	1	0.6-1.4	mg/dL
eGFR (CKD-EPI 2021)	76	≥90	mL/min/1.73 m²
Sodium	138	133-145	mEq/L
Potassium	4.7	3.3-5.1	mEq/L
Chloride	102	95-108	mEq/L
CO₂ (bicarbonate)	23	21-30	mEq/L
Anion Gap	18	14-20	mEq/L
Calcium	9.6	8.3-10.5	mg/dL
AST (SGOT)	21	0-40	IU/L
ALT (SGPT)	29	0-41	IU/L
Alkaline phosphatase	73	35-129	IU/L
Total bilirubin	0.6	0-1.2	mg/dL
Total protein	8.3	6.4-8.3	g/dL
Albumin	4.3	3.5-5.2	g/dL

**Figure 1:**
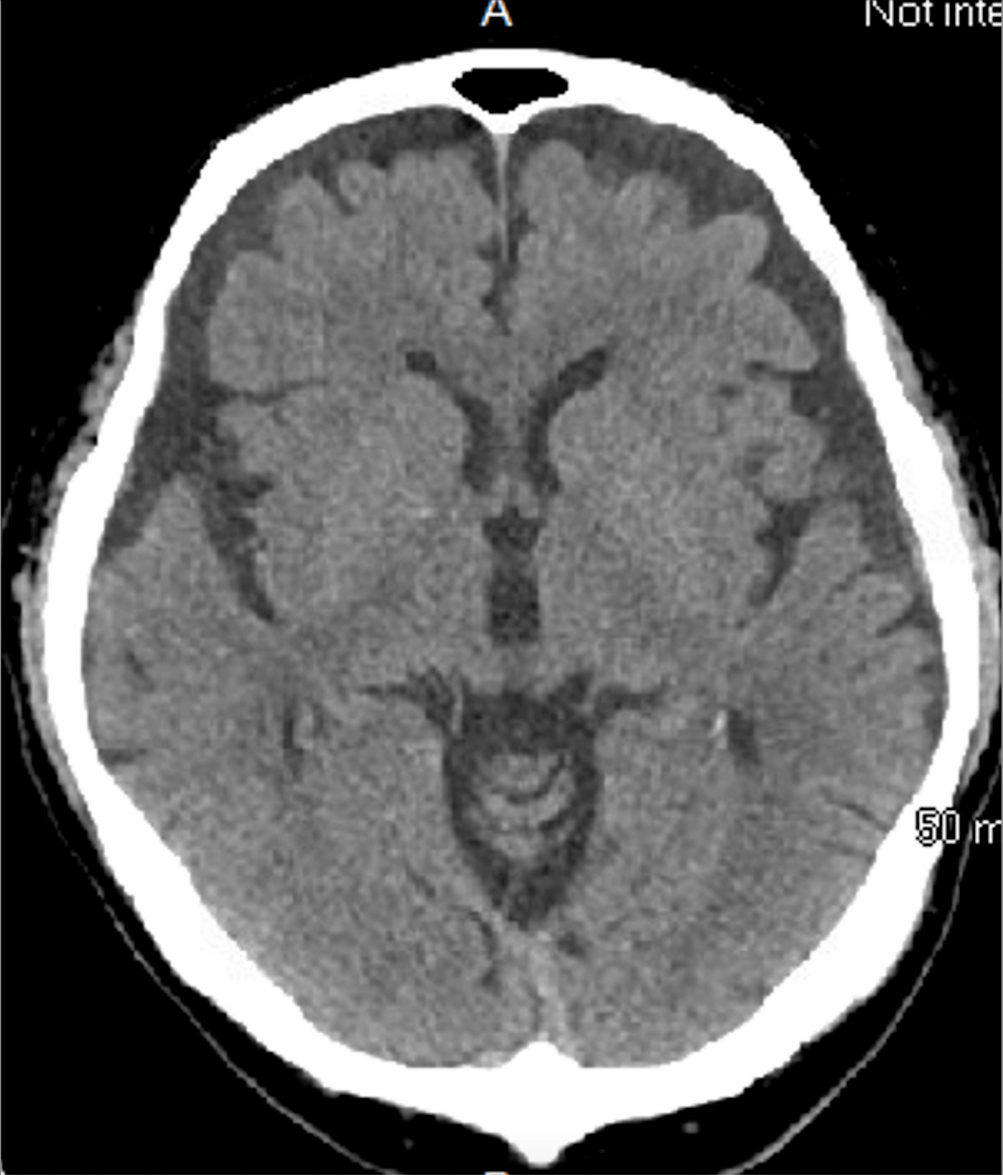
Non-contrast head CT scan showing no acute abnormalities

**Figure 2:**
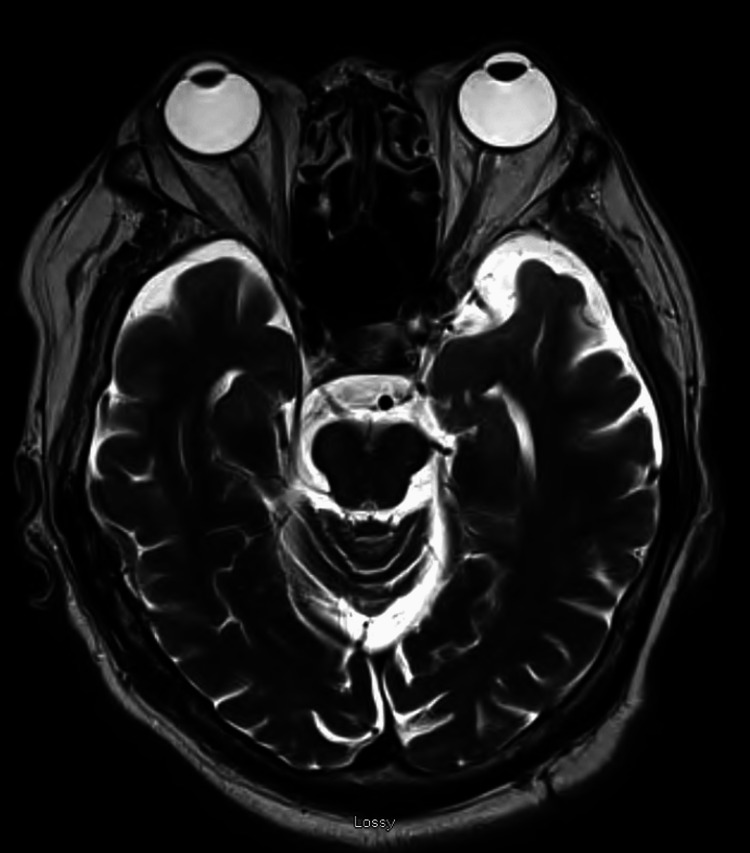
MRI of the brain demonstrating normal findings

**Figure 3:**
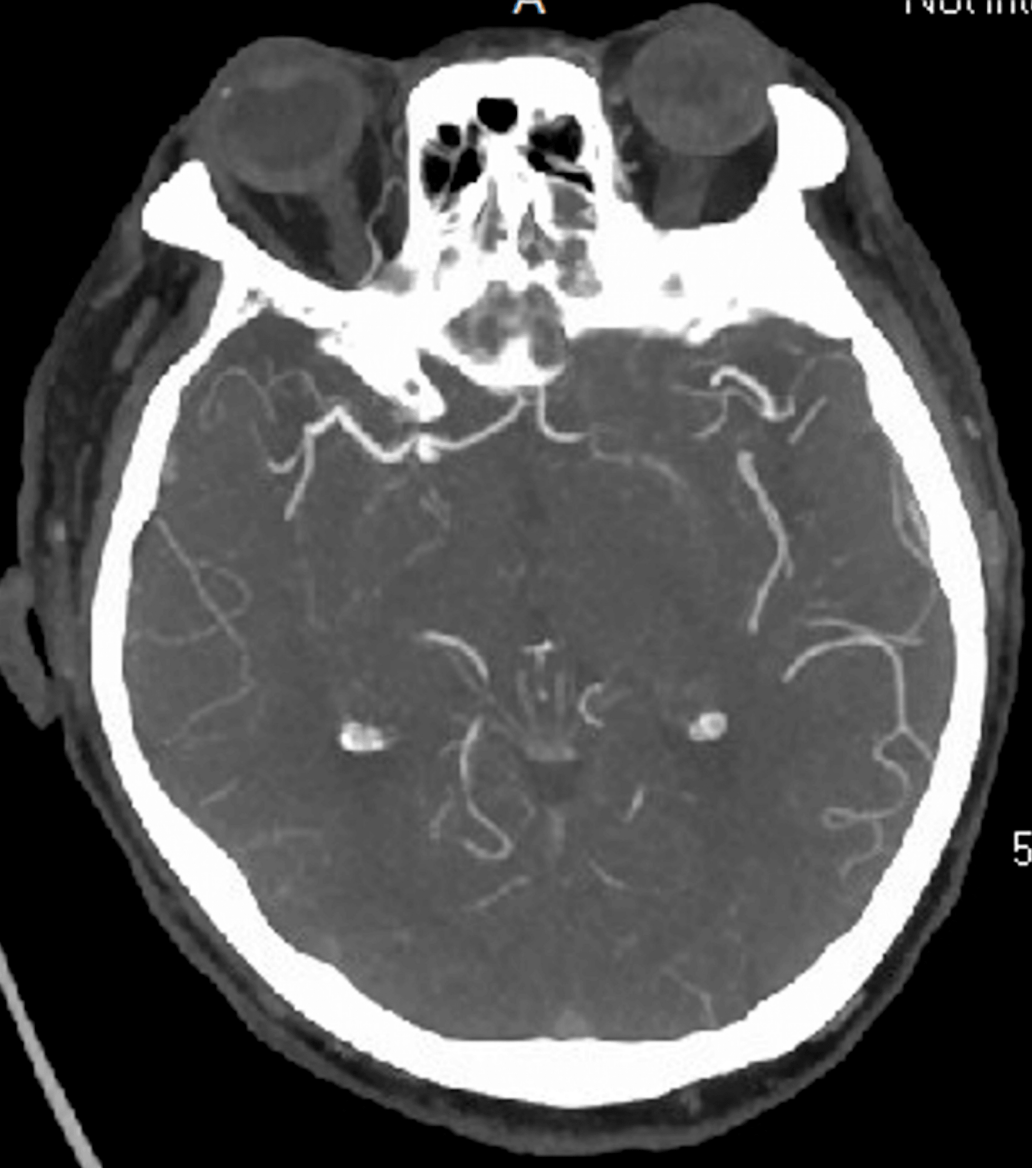
CT angiogram of the head revealing no evidence of vascular abnormalities

Non-contrast head CT (Figure 1), CT angiography of the head and neck (Figure 2), and brain MRI (Figure 3) were unremarkable, showing no evidence of infarcts, hemorrhage, mass lesions, or demyelinating changes. Lumbar puncture yielded clear CSF with normal opening pressure and no albuminocytologic dissociation (Table 3).

**Table 3:** CSF analysis

Test	Result	Reference range	Units
CSF color	Colorless	Colorless	-
CSF appearance	Clear	Clear	-
CSF supernatant	Colorless/clear	Colorless	-
CSF RBC	143	0	cells/µL
CSF WBC	8	0-5	cells/µL
CSF protein	24	15-45	mg/dL
CSF glucose	78	40-70	mg/dL
CSF Gram stain	No organisms seen	No cells	-
CSF culture	No growth after five days	No growth	-

Of note, anti-GQ1b IgG antibodies were strongly positive, with titers exceeding 1:12,800 (Table 4). Based on the clinical presentation and serologic findings, a diagnosis of atypical MFS was established, characterized by isolated ophthalmoplegia in the absence of areflexia, ataxia, or albuminocytologic dissociation. The patient was initiated on IVIG therapy at a dose of 0.4 g/kg/day for five consecutive days. He tolerated the treatment well and experienced no complications.

**Table 4:** Inflammatory, endocrine, autoimmune, complement, infectious, and nutritional laboratory findings Ab, antibody; ACE, angiotensin-converting enzyme; ANA, antinuclear antibody; ANCA, antineutrophil cytoplasmic antibody; Anti-dsDNA, anti–double-stranded DNA antibody; CK, creatine kinase; CRMP-5, collapsin response mediator protein 5; ESR, erythrocyte sedimentation rate; Free T4, free thyroxine; GD1a/1b Ab, anti-GD1a/GD1b ganglioside antibody; GM1 Ab, anti-GM1 ganglioside antibody; GQ1b Ab, anti-GQ1b ganglioside antibody; HIV, human immunodeficiency virus; MAG Ab, myelin-associated glycoprotein antibody; MOG FACS, myelin oligodendrocyte glycoprotein by fluorescence-activated cell sorting; NMO/AQP4 FACS, neuromyelitis optica / aquaporin-4 by fluorescence-activated cell sorting; RA, rheumatoid arthritis; RPR, rapid plasma reagin; SSB/La, Sjögren’s syndrome-related antigen B (La); SSA/Ro, Sjögren’s syndrome-related antigen A (Ro); TSH, thyroid-stimulating hormone; VGKC, voltage-gated potassium channel.

Test	Result	Reference range	Units
CRP	11.7 ↑	0.0-10.0	mg/L
ESR	54 ↑	0-20	mm/hr
CK, total	113	39-308	IU/L
Vitamin B1	117	78-185	nmol/L
Vitamin B1 (repeat)	163	78-185	nmol/L
RPR	Nonreactive	Nonreactive	-
Cryoglobulins	Negative	Negative	-
ANA	Negative	Negative	-
ANCA	Negative	Negative	-
Anti-dsDNA	<1	<1	-
Anti-SSA/Ro	<0.2	≤0.9 Negative	-
Anti-SSB/La	<0.2	≤0.9 Negative	-
C3 complement	135	90-180	mg/dL
C4 complement	34	10-40	mg/dL
ACE	5 ↓	9-67	-
MOG FACS	Negative	Negative	-
NMO/AQP4 FACS	Negative	Negative	-
Striated Muscle Ab	Negative	Negative	-
Anti-glial nuclear Ab	Negative	Negative	-
Amphiphysin Ab	Negative	Negative	-
Anti-neuronal nuclear Ab type 1	Negative	Negative	-
Anti-neuronal nuclear Ab type 2	Negative	Negative	-
Anti-neuronal nuclear Ab type 3	Negative	Negative	-
P/Q calcium channel Ab	0	-	nmol/L
CRMP-5 IgG	Negative	Negative	-
Purkinje cell Ab type 1	Negative	Negative	-
Purkinje cell Ab type 2	Negative	Negative	-
Purkinje cell Ab type Tr	Negative	Negative	-
Neuronal VGKC Ab	0	-	nmol/L
Hepatitis B surface antigen	Negative	Negative	-
Hepatitis B core Ab (IgG/IgM)	Negative	Negative	-
Hepatitis B surface Ab	Negative	Negative	-
Hepatitis C antibody	Negative	Negative	-
HIV antigen/antibody	Negative	Negative	-
TSH	1.36	0.4-4.5	µIU/mL
Free T4	1.1	0.8-1.8	ng/dL
Rheumatoid factor (RA)	<10	<14	IU/mL
GQ1b Ab (IgG)	>1:12,800 ↑	<1:100	Titer
GM1 Ab (IgG)	<1:800	<1:800	Titer
GM1 Ab (IgM)	<1:800	<1:800	Titer
GD1b Ab (IgM)	<1:800	<1:800	Titer
Asialo-GM1 Ab (IgM)	<1:1600	<1:1600	Titer
GD1a Ab (IgG)	<1:100	<1:100	Titer
GD1a Ab (IgM)	<1:800	<1:800	Titer
GD1b Ab (IgG)	<1:100	<1:100	Titer
Asialo-GM1 Ab (IgG)	<1:100	<1:100	Titer
MAG Ab (IgM) Western blot	Negative	Negative	-
Hu Ab screen	Negative	Negative	-

At follow-up one week after discharge, the patient reported complete resolution of diplopia. Neurologic examination confirmed full recovery of extraocular muscle function, and he remained symptom-free with no recurrence at subsequent outpatient visits.

## Discussion

GBS is an immune-mediated polyradiculopathy and is the leading cause of acute flaccid paralysis globally [5,6]. Among its variants, MFS is characterized by the triad of ophthalmoplegia, ataxia, and areflexia. The global incidence of GBS is approximately 1-2 per 100,000 annually, whereas MFS represents a much smaller proportion, estimated at 1-2 cases per million [7,8]. MFS is more common in men, with a male-to-female ratio of 2:1 and a mean age of onset of 43.6 years. Its prevalence is higher in Asia (15-25% of GBS cases) compared to Western countries (1-7%) [7,9-11].

The classic MFS triad - ophthalmoplegia, ataxia, and areflexia - is seen in about 80% of cases. Similar to GBS, MFS is typically preceded by an infection. The interval between the preceding infection and the onset of neurological symptoms typically ranges from eight to 10 days but may vary from one to 30 days [7,12]. Diplopia is often the initial symptom, followed by the gradual development of ophthalmoplegia and ataxia, with areflexia occurring later in the disease course [7,10]. Less frequent symptoms accompanying the triad may include limb dysesthesias, ptosis, and cranial nerve involvement affecting the face, bulbar muscles, or pupils [7,11,13].

The diagnosis of MFS is supported by the clinical triad, a history of antecedent infection, and the presence of anti-ganglioside antibodies, most notably anti-GQ1b IgG, which is present in over 90% of patients and is highly specific for the condition [12-14]. Beyond the classic triad, atypical presentations of MFS have been reported in the literature [15]. These include bilateral internal ophthalmoplegia, unilateral oculomotor nerve paralysis, severe bilateral headache, and partial Parinaud’s syndrome, highlighting the clinical variability of the disorder.

Our case, featuring isolated bilateral ophthalmoplegia without ataxia or areflexia, represents an atypical variant of MFS. Such cases fall within the broader spectrum of anti-GQ1b antibody syndromes and have been described in previous literature. Due to the rarity of MFS and its typically self-limiting nature, limited data exist regarding treatment, and no randomized controlled trials have been published to date [7,14,16].

In our patient, laboratory abnormalities such as an elevated ESR (54 mm/hr) and mildly increased CRP (11.7 mg/L) suggested an underlying inflammatory process. Neuroimaging (brain MRI and CT angiography) was normal, ruling out central causes like stroke or mass lesions. Although CSF analysis did not show albuminocytologic dissociation, differential diagnoses - including myasthenia gravis, brainstem stroke, Wernicke’s encephalopathy, and paraneoplastic syndromes - were excluded based on imaging, serologic results, preserved mental status, and the absence of malignancy. While the classic MFS triad was not fully present, our findings support an atypical variant of MFS with isolated ophthalmoplegia following viral conjunctivitis.

The pathophysiology of MFS involves molecular mimicry following infection, leading to autoimmune targeting of ganglioside components in peripheral nerves. Among the anti-ganglioside antibodies, anti-GQ1b plays a pivotal role. It is not only a reliable diagnostic marker but also correlates with disease severity and progression [17]. GQ1b ganglioside is found predominantly in cranial nerves (oculomotor, abducent, and trochlear) and in the dorsal root ganglia [6,18]. The binding of anti-GQ1b IgG to these sites triggers complement-mediated injury and conduction block, resulting in ophthalmoplegia, proprioceptive dysfunction (ataxia), and areflexia. The high concentration of GQ1b in the oculomotor nerve explains the frequent involvement of ocular motility in MFS [17].

Other implicated anti-ganglioside antibodies in MFS include anti-GM1, anti-GD1a, anti-GD1b, anti-GT1a, anti-LM1, and anti-GD3 [17,19]. Atypical MFS presentations may be due to diverse antibody reactivities beyond GQ1b [9]. Serum antibody profiles vary among patients; some have antibodies against both GQ1b and GM1, but not GQ1b or GT1a alone. These antibody differences may account for the varied clinical manifestations. For instance, sensory deficits and paresthesias are uncommon in patients with both GQ1b and GM1 reactivity but more frequent in those with antibodies specific to GQ1b or GT1a. Furthermore, patients lacking ophthalmoplegia or other motor symptoms may possess antibodies to GD1b and/or GD3 rather than to GQ1b or GT1a [19,20]. Thus, the clinical features of MFS are likely influenced by the specific profile of anti-ganglioside antibodies and the anatomical distribution of their target antigens.

A prospective case series from Japan found that IVIG led to earlier symptom resolution, while plasmapheresis did not significantly hasten recovery [16]. IVIG may facilitate clinical improvement by blocking anti-GQ1b binding and preventing complement activation and subsequent nerve damage. This case highlights the importance of early recognition of atypical MFS presentations and the need for a comprehensive evaluation to ensure appropriate management and prevent complications. Prompt diagnosis and confirmation with anti-GQ1b testing are essential for guiding timely and effective treatment.

## Conclusions

MFS can present with atypical features, including isolated ophthalmoplegia without ataxia, areflexia, or CSF abnormalities. This case underscores that high anti-GQ1b antibody titers are a key diagnostic indicator, even when the full clinical triad is absent. Clinicians should consider this diagnosis in post-viral neurologic syndromes involving selective cranial nerve dysfunction. Early recognition and treatment with IVIG can result in rapid recovery and excellent outcomes.
